# A feeder-free culture using autogeneic conditioned medium for undifferentiated growth of human embryonic stem cells: Comparative expression profiles of mRNAs, microRNAs and proteins among different feeders and conditioned media

**DOI:** 10.1186/1471-2121-11-76

**Published:** 2010-10-12

**Authors:** Zong-Yun Tsai, Sher Singh, Sung-Liang Yu, Chi-Hsien Chou, Steven Shoei-Lung Li

**Affiliations:** 1 Stem Cell Laboratory, Center of Excellence for Environmental Medicine, Kaohsiung Medical University, Kaohsiung 807, Taiwan; 2 Department of Life Science, College of Science, National Taiwan Normal University, Taipei 116, Taiwan; 3 Department of Clinical Laboratory Sciences and Medical Biotechnology, National Taiwan University College of Medicine, Taipei 100, Taiwan; 4 Center for Resources, Research and Development, Kaohsiung Medical University, Kaohsiung 807, Taiwan; 5 Graduate Institute of Clinical Medicine, College of Medicine, Kaohsiung Medical University, Kaohsiung 807, Taiwan; 6 Correspondence to: Steven Shoei-Lung Li at Graduate Institute of Clinical Medicine, College of Medicine, Kaohsiung Medical University, Kaohsiung 807, Taiwan

## Abstract

**Background:**

Human embryonic stem (hES) cell lines were derived from the inner cell mass of human blastocysts, and were cultured on mouse embryonic fibroblast (MEF) feeder to maintain undifferentiated growth, extensive renewal capacity, and pluripotency. The hES-T3 cell line with normal female karyotype was previously used to differentiate into autogeneic fibroblast-like cells (T3HDF) as feeder to support the undifferentiated growth of hES-T3 cells (T3/HDF) for 14 passages.

**Results:**

A feeder-free culture on Matrigel in hES medium conditioned by the autogeneic feeder cells (T3HDF) was established to maintain the undifferentiated growth of hES-T3 cells (T3/CMHDF) for 8 passages in this investigation. The gene expression profiles of mRNAs, microRNAs and proteins between the undifferentiated T3/HDF and T3/CMHDF cells were shown to be very similar, and their expression profiles were also found to be similar to those of T3/MEF and T3/CMMEF cells grown on MEF feeder and feeder-free Matrigel in MEF-conditioned medium, respectively. The undifferentiated state of T3/HDF and T3/CMHDF as well as T3/MEF andT3/CMMEF cells was evidenced by the very high expression levels of "stemness" genes and low expression levels of differentiation markers of ectoderm, mesoderm and endoderm in addition to the strong staining of OCT4 and NANOG.

**Conclusion:**

The T3HDF feeder and T3HDF-conditioned medium were able to support the undifferentiated growth of hES cells, and they would be useful for drug development and toxicity testing in addition to the reduced risks of xenogeneic pathogens when used for medical applications such as cell therapies.

## Background

Human embryonic stem (hES) cell lines were derived from the inner cell mass of human blastocysts, and were cultured on mouse embryonic fibroblast (MEF) feeder to maintain undifferentiated growth, extensive renewal capacity, and pluripotency, including the ability to form teratomas in SCID mice and embryoid bodies *in vitro *[1]. The hES cells were later shown to be able to retain their fundamental characteristics by culturing on Matrigel in MEF-conditioned medium, and this feeder-free culture system is suitable for scale up production of undifferentiated hES cells [2]. In addition to their contribution to basic research such as stem cell biology and early human development, hES cells have great potential as source of cells for therapeutic uses. In order to reduce the risks of cross-transfer of pathogens from xenogeneic feeder or conditioned medium, an autogeneic feeder cell system, comprising fibroblast-like cells differentiated from hES cells, was developed to grow undifferentiated and pluripotent hES cells for their medical applications [3]. A feeder-free culture using medium conditioned by autogeneic feeder cells is desirable in order to use hES cells as tools for drug development and toxicity testing.

In our laboratory, five hES cell lines had been derived [4], and one line hES-T3 with normal female karyotype was used to establish autogeneic feeder cells with capacity to support the growth of undifferentiated hES cells [5]. In this investigation, a feeder-free culture on Matrigel in medium conditioned by these autogeneic feeder cells was established to maintain the undifferentiated growth of hES cells, and the gene expression profiles of mRNAs, microRNAs (miRNAs) and proteins were further shown to be very similar between the undifferentiated hES cells grown on autogeneic feeder and its conditioned medium, as well as MEF feeder and MEF-conditioned medium.

## Methods

### Undifferentiated growth of hES cells on MEF feeder and MEF-conditioned medium

Human embryonic stem cell line hES-T3, which is one of the five hES cell lines derived in our laboratory with institutional review board approval and informed consent by couples undergoing IFV treatment in Taiwan [4], exhibits normal female karyotype (46, XX), and it has been continuously cultured on mitomycin C (10 ug/ml) mitotically inactivated MEF feeder in hES medium under 5% CO_2 _at 37°C and underwent freezing/thawing processes. The hES culture medium consisted of DMEM/F12 (1:1, GIBCO) supplemented with 20% KSR (Invitrogen), 1% non-essential amino acids, 1 mM L-glutamine, 0.1 mM β-mercaptoethanol, and 4 ng/ml human basic fibroblast growth factor (bFGF; Life Technologies). Routine passages of hES-T3 cells every 5-7 days were done with collagenase (type IV, 1 mg/ml, Invitrogen) treatment and mechanical scrape [4,5]. The cryopreserved stock of hES-T3 cells (36 passages) were continuously maintained on MEF feeder for additional 14 passages, and these the hES-T3 cells were designated as T3/MEF [6].

The MEF cells were cultured in MEF medium overnight, and the mitotically inactivated MEF cells were maintained in hES medium containing 4 ng/ml bFGF. After 24 h, the MEF-conditioned medium was collected and filtered through 0.2 um membrane (PN4612, Pall Life Sciences) as previously described [2]. The culture dish was coated with Matrigel diluted with DMEM/F12 (1:30) overnight at 4°C. The cryopreserved stock of hES-T3 cells (36 passages) were continuously maintained on feeder-free Matrigel-coated dish in MEF-conditioned medium (with additional 4 ng/ml bFGF) for 12 passages, and these hES-T3 cells were designated as T3/CMMEF [6].

### Establishment of human hES-T3 differentiated fibroblast-like cells

Autogeneic feeder cells with capacity to support the growth of undifferentiated hES cells were established according to the previously published procedure [3]. hES-T3 (passage 19) cells were transferred into feeder-free and noncoated plate (10 cm) in DMEM supplemented with 10% FBS (GIBCO) under 5% CO_2 _at 37°C. After 10 days, cells appeared as fibroblast-like morphology, that is, flat cells with elongated nucleus and branching pseudopodia. These hES-T3 differentiated fibroblast-like cells are designated as T3HDF. The expression of transcription factors OCT4, SOX2 and NANOG, which were highly expressed in T3/MEF cells, was shown to be down-regulated in differentiated T3HDF cells. The expression profiles of mRNAs and miRNAs between T3/MEF and T3HDF cells were also found to very different [5]. These T3HDF cells were passaged using trypsin (0.05%, GIBCO) every 4 days or cryopreserved.

### Undifferentiated growth of hES cells on T3HDF feeder and T3HDF-conditioned medium

The differentiated fibroblast-like T3HDF cells (passage 8) were inactivated using mitomycin C (10 ug/ml) and used as autogeneic feeder layer in hES medium to maintain the continuously undifferentiated growth of hES-T3 cells (36 passages on MEF) for additional 14 passages [5]. These hES-T3 cells grown on T3HDF feeder were designated as T3/HDF.

The T3HDF cells were cultured in DMEM medium overnight, and the mitotically inactivated T3HDF were maintained in hES medium containing 4 ng/ml bFGF. After 24 h, the T3HDF-conditioned medium was collected and filtered through 0.2 um membrane [2]. The culture dish was coated with Matrigel diluted with DMEM/F12 (1:30) overnight at 4°C. The hES-T3 cells (36 passages on MEF feeder) were first grown on T3HDF feeder for 4 passages and then on Matrigel in T3HDF-conditioned medium for additional 4 passages. The hES-T3 cells grown on feeder-free Matrigel-coated dish in T3HDF-conditioned medium (with additional 4 ng/ml bFGF) were designated as T3/CMHDF

### Staining of OCT4 and NANOG

T3/HDF and T3/CMHDF, as well as T3/MEF and T3/CMMEF, colonies were fixed by 4% paraformaldehyde and permeabilized using 0.5% Triton X-100 in the culture dishes. The immunostaining with rabbit polyclonal antibodies against human OCT4 (POU5F1) and NANOG (Santa Cruz Biotechnology) were detected with goat anti-rabbit IgG as described previously [5,6].

### Extraction of total RNAs

Total RNAs from approximately 1 × 10^6 ^cells of T3/HDF and T3/CMHDF on 10 cm plate were extracted using TRIZOL reagent, and the same total RNAs from each sample were used for both mRNA microarray analysis and miRNA quantification.

### mRNA microarray analysis

The mRNA profilings of T3/HDF and T3/CMHDF cells were analyzed using Affymetrix Human Genome U133 plus 2.0 GeneChip according to the Manufacturer's protocols (Santa Clara, CA, USA, http://www.affymetrix.com) by the Microarray Core Facility of National Research Program for Genomic Medicine of National Science Council in Taiwan as previously described [5,6]. This Affymetrix GeneChip contains 54,675 probe sets to analyze the expression levels of 47,400 transcripts and variants, including 38,500 well-characterized human genes. GeneChips from the hybridization experiments were read by the Affymetrix GeneChip scanner 3000, and raw data were processed using Affymetrix GeneChip Operating Software MAS5.0 and its default analysis parameters. The raw data were also analyzed by GeneSpring GX software version 7.3.1 (Silicon Genetics, Redwood City, CA, USA, http://www.chem.agilent.com). The correlation coefficients of gene probes expressed between any two samples were calculated from the normalized values by using GeneChip-Robust Multiarray Average (GC-RMA) algorithm. It may be noted that Affymetrix GeneChip expression analysis can be used as a stand-alone quantitative comparison, since the correlation between Affymetrix GeneChip results and TagMan RT-qPCR results was shown in a good linearity of R^2 ^= 0.95 by the MicroArray Quality Control Study, a collaborative effort of 137 scientists led by the US-FDA [7,8]. A hierarchical clustering and principle component analysis (PCA) of the eight Affymetrix GeneChip data from duplicates of four populations of hES cells were also performed in order to check the quality of microarray results.

### Analyses of signaling pathways and GO process networks

The abundantly (more than 3-folds of overall mean) expressed mRNAs of T3/HDF and T3/CMHD, as well as T3/MEF and T3/CMMEF, cells were analyzed for signaling pathways and GO process networks by using MetaCore Analytical Suite (GeneGo Inc., St Joseph, MI, USA) as previously described [6]. The MetaCore includes a curated database of human protein interaction and metabolism, and thus it is useful for analyzing a cluster of genes in the context of regulatory network and signaling pathways.

### Quantification of miRNAs

The expression levels of 365 human miRNAs from T3/HDF and T3/CMHD cells were determined using the TaqMan MicroRNA Assays (Applied Biosystems, Foster City, California, USA, http://www.appliedbiosystems.com) [9,10]. The detailed procedure for miRNA quantification was previously described [5,6]. In brief, TagMan MicroRNA Assays include two steps: stem loop RT followed by real-time PCR. (90 ng/Rx, with 24-multiplex primers) Each 10 ul RT reaction that includes 90 ng total RNA, 50 nM stem-loop RT primers, 1× RT buffer, 1.25 mM each of dNTPs, 0.25 U/ul RNase inhibitor, and 10 U/ul MultiScribe Reverse Transcriptase was incubated in the PTC-225 Peltier Thermal Cycler (MJ Research, Watertown, Massachusetts, USA) for 30 min each at 16°C and at 42°C, followed by 5 min at 85°C, and then held at 4°C. RT products were diluted twenty times with H_2_O prior to setting up PCR reaction. Real-time PCR for each miRNA was carried out in triplicates, and each 10 ul reaction mixture included 2 ul of diluted RT product, 5 ul of 2× TagMan Universal PCR Master Mix and 0.2 uM TagMan probe, respectively. The reaction was incubated in an Applied Biosystems 7900HT Sequence Detection System at 95°C for 10 min, followed by 40 cycles of 95°C for 15 sec and 60°C for 1 min. The threshold cycle (Ct) is defined as the fraction cycle number at which the fluorescence exceeds the fixed threshold of 0.2. Total RNA input was normalized based on the Ct values of the TagMan U6 snRNA assay as an endogenous control. The fold change was calculated as 2^-ΔCT ^× K, where -ΔCT = -[CT_miRNA_-CT_U6 snRNA_] and K is a constant.

### 2D-gel analysis of proteins

Approximately 1 × 10^6 ^hES cells on 10 cm plate were washed twice each with 1× PBS and cell wash buffer, and then lyzed using NP40 lysis buffer. 1 mL ice-cold acetone/11% w/v trichloroacetic acid (TCA)/20 mM DTT was added *per *0.1 mL solubilised sample and incubated for a minimum of 30 min at -20°C. The precipitate was pelleted by centrifugation (12000 rpm for 10 min at 4°C), washed twice with 1 mL cold acetone containing 20 mM DTT, and then air-dried to remove residual acetone. The resulting protein pellet was then resolubilised in the appropriate rehydration buffer (7 M urea, 2 M thiourea, 2% CHAPS, 0.5% IPG buffer, 20 mM DTT). The concentration of proteins in the sample was measured by the Bradford method.

Isoelectricfocusing was performed using an Ettan IPGphor II (Amersham Biosciences). 13 cm Immobiline DryStrips (pH 3-10 NL) were rehydrated overnight for 12 h at room temperature in 250 uL rehydration buffer containing 7 M urea, 2 M thiourea, 2% w/v CHAPS, 20 mM DTT, 0.5% IPG buffer and a trace of bromophenol blue. The protein sample (about 100 ug) was mixed in 100 uL sample buffer containing 7 M urea, 2 M thiourea, 2% CHAPS, 0.5% IPG buffer pH 3-10 NL, 100 mM DeStreak reagent (Amersham biosciences) and a trace of bromophenol blue. Samples were cup-loaded near the anode of the IPG strips using Ettan IPGphor cup-loading (Amersham Biosciences) according to the manufacturer's protocol. Protein focusing was achieved using the following IEF parameters: 300 V, step and hold, 3 h; 600 V, gradient, 1 h; 1000 V, gradient, 1 h; 8000 V, gradient, 1.5 h; 8000 V, step and hold for 3 h, giving a total of 16000 Vh.

After focusing, the strips were removed immediately and equilibrated by gentle shaking for 15 min in 10 mL equilibration buffer (50 mM Tris-base, pH = 8.8, 6 M urea, 30% v/v glycerol, 0.2% w/v SDS and 1% w/v DTT), followed by 10 mL of the same solution containing 2.5% w/v iodoacetamine instead of DTT for 15 min. The second dimension was performed by SDS-polyacrylamide gel electrophoresis (PAGE) on a 12% w/v separation gel using the Hoefer SE 600 vertical chambers. First dimension IPG gel strips were cut and placed on top of the second dimension vertical gels (16 × 18 × 0.01 cm) and sealed in place with boiling 0.5% agarose in running buffer, containing 0.025 M Tris base, 0.192 M glycine, 0.1% w/v SDS, pH 8.3. The second dimension separation was performed sequentially with a constant voltage of 70 V for 0.5 h, and 120 V for 12 h. After SDS-PAGE, the separated gels were visualized by silver staining. The similarities of protein spots on scanned images were analyzed using ImageMaster 2DE platinum software version 5.0 (Amersham Biosciences).

## Results

### Characterization of undifferentiated T3/HDF and T3/CMHDF cells

The hES-T3 cells (36 passages on MEF feeder) were cultured on T3HDF feeder in hES medium (containing 4 ng/ml bFGF) (T3/HDF) and feeder-free Matrigel in T3HDF-conditioned medium with additional 4 ng/ml bFGF (T3/CMHDF) for 14 and 8 passages, respectively. The T3/HDF and T3/CMHDF, as well as T3/MEF and T3/CMMEF, cells were stained positively for OCT4 and NANOG (Additional file 1: Fig. S1).

### Expression profiling of mRNAs

**Figure 1:**
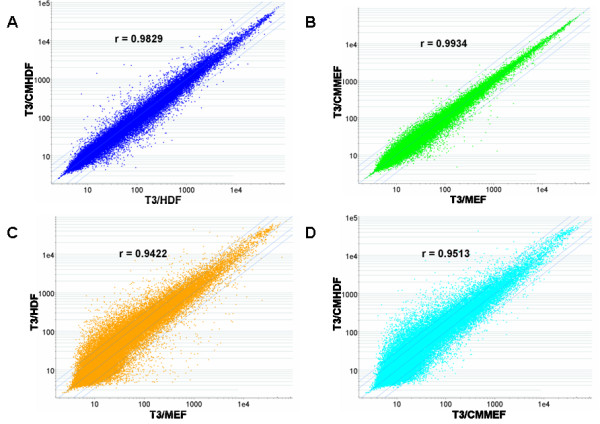
**Scatter plots and correlation analyses of mRNAs among T3/HDF, T3/CMHDF, T3/MEF and T3/CMMEF cells**. A. T3/HDF vs T3/CMHDF; B. T3/MEF vs T3/CMMEF; C. T3/MEF vs T3/HDF; D. T3/CMMEF vs T3/CMHDF.

The genome-wide mRNA expression profiles of T3/HDF and T3/CMHDF cells were determined using Affymetrix human genome U133 plus 2.0 GeneChip. The original data have been deposited to NCBI database, and the GEO series number is GSE19902. The average values of duplicate analyses for expressed mRNAs from T3/HDF and T3/CMHDF cells were compared by scatter plot (Fig. 1A). The Pearson correlation coefficient of r = 0.9829 between T3/HDF and T3/CMHDF cells indicates their very similar expression profiles of mRNAs, and only 102 and 84 genes were found to be abundantly (more than 3-folds of overall mean) differentially (more than 3-folds of changes) expressed in T3/HDF and T3/CMHDF cells, respectively (Additional files 2, 3: Table S1A,B). It may be noted that galanin and galectin 1 were the most abundant and expressed at extremely high levels of 793 and 1276 folds of overall mean in T3/HDF and T3/CMHDF cells, respectively.

The mRNA expression profiles of T3/HDF and T3/CMHDF cells were also compared with those (GSE9440) of T3/MEF and T3/CMMEF cells determined previously [6] in Fig. 1, and very high similarities were found among these four populations of hES-T3 cells, that is, the values of r = 0.9934 between T3/MEF and T3/CMMEF, r = 0.9422 between T3/MEF and T3/HDF, r = 0.9513 between T3/CMMEF and T3/CMHDF cells. It may be noted that hierarchical clustering and principle component analysis (PCA) of all GeneChip results from four hES cell populations indicated the duplicate data were closely related, implying the good quality of their microarray data (Additional file 4: Fig. S2). The very high expression levels of 21 "stemness" genes such as OCT4 (POU5F1) and NANOG, as well as low expression levels of 9 differentiation markers of ectoderm, mesoderm and endoderm [4], from T3/HDF, T3/CMHDF, T3/MEF and T3/CMMEF cells (Table 1) indicate that these four cell populations contained very high proportions of undifferentiated hES cells. The fold-changes of the 21 "stemness" genes and 9 differentiation markers among these four cell populations (Additional file 5: Table S2) indicate that SALL4 gene appeared to express much higher level in T3/HDF cells compared with other three cell populations.

**Table 1:** The expression levels of "stemness" genes and differentiation markers.

A. 21"stemness" genes							
Probe ID	Gene Symbol	T3/HDF	T3/CMHDF	T3/MEF	T3/CMMEF	UniGene	Gene Description
206286_s_at	TDGF1	638.00	555.60	325.10	352.70	Hs.385870	Teratocarcinoma-derived growth factor 1, CRIPTO
220184_at	NANOG	586.80	674.20	614.80	680.70	Hs.329296	Nanog homeobox
210905_x_at	ASH1L	331.30	335.60	196.10	197.80	Hs.491060	ASH1L (Drosophila), hypothetical protein, 2969aa
210265_x_at	POU5F1L	186.10	176.50	236.80	221.70	Hs.504658	Similar to POU domain Class 5 transcription factor 1
219651_at	DPPA4	126.40	137.90	92.07	116.50	Hs.317659	Developmental pluripotency associated 4, 294aa
224048_at	USP44	94.35	81.71	43.63	44.44	Hs.506394	Ubiquitin specific protease 44
229661_at	SALL4	87.55	15.74	4.45	2.93	Hs.517113	Sal-like 4 (Drosophila), zinc finger protein
208286_x_at	POU5F1	87.31	79.01	86.01	92.14	Hs.249184	POU domain Class 5 Transcription Factor 1,OCT4, 360aa
206309_at	LECT1	66.61	62.77	25.02	38.87	Hs.421391	Leukocyte cell derived chemotaxin 1
214532_x_at	POU5F1P1	65.41	63.29	47.31	56.49	Hs.450254	POU domain, OCT4-related intron-less gene, 359aa
240301_at	DPPA2	55.90	119.20	27.86	43.06	Hs.351113	Developmental pluripotency associated 2, 221aa
206023_at	NMU	54.32	91.65	44.98	40.92	Hs.418367	Neuromedin U
214829_at	AASS	30.42	21.40	7.60	9.77	Hs.528295	Aminoadipate-semialdehyde synthase
213050_at	COBL	23.24	19.65	3.35	5.52	Hs.99141	Cordon-bleu homolog (mouse)
208542_x_at	ZNF208	14.12	10.08	2.94	3.97	Hs.419763	Zinc finger protein 208
231698_at	UGP2	8.20	12.80	8.31	10.50	Hs.516217	UDP-glucose pyrophosphorylase 2
205309_at	SMPDL3B	5.88	2.90	4.68	2.95	Hs.123659	Acid sphingomyelinase-like phosphodiesterase
202889_x_at	MAP7	5.44	6.46	5.38	5.18	Hs.486548	Microtubule-associated protein 7
230623_x_at	USP28	5.12	8.38	2.81	4.55	Hs.503891	Ubiquitin specific protease 28
215509_s_at	BUB1	4.90	2.29	3.45	2.33	Hs.469649	BUB1 budding (yeast), human spindle check point kinase
207199_at	TERT	4.03	4.06	3.27	2.24	Hs.492203	Telomerase reverse transcriptase

**B. 9 differentiation markers**							
**Probe ID**	**Gene Symbol**	**T3/HDF**	**T3/CMHDF**	**T3/MEF**	**T3/CMMEF**	**UniGene**	**Gene Description**

**Ectoderm**							
225540_at	**MAP2**	0.12	0.13	0.59	0.61	Hs.368281	microtubule-associated protein 2
1556057_s_at	**NEUROD1**	0.54	0.56	0.67	0.68	Hs.709709	neurogenic differentiation 1
1555938_x_at	**VIM**	0.17	0.19	0.52	0.40	Hs.642813	vimentin
**Mesoderm**							
205932_s_at	**MSX1**	0.09	0.07	0.10	0.07	Hs.424414	msh homeobox 1
202222_s_at	**DES**	0.63	0.67	0.68	0.63	Hs.594952	desmin
1556499_s_at	**COL1A1**	0.49	0.19	0.20	0.34	Hs.172928	collagen, type I, alpha 1
**Endoderm**							
224646_x_at	**H19**	0.58	0.75	0.97	1.00	Hs.533566	H19, imprinted maternally untranslated mRNA
214701_s_at	**FN1**	0.08	0.07	0.41	0.16	Hs.203717	fibronectin 1
211896_s_at	**DCN**	0.01	0.02	0.01	0.03	Hs.706840	decorin
							

### Signaling pathways and GO process networks

**Figure 2:**
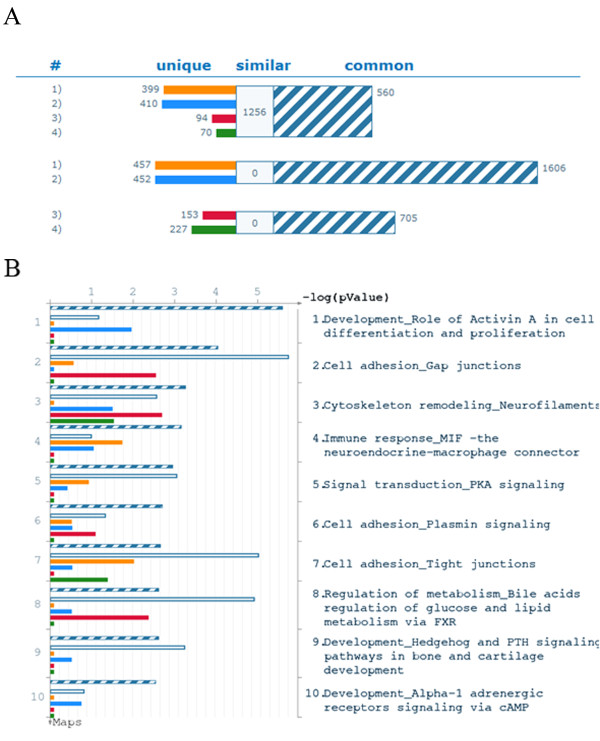
**Comparison of gene expression and GeneGo canonical pathway maps among T3/HDF, T3/CMHDF, T3/MEF and T3/CMMEF cells**. A. Parameters for comparison are set at threshold of 3 with p-value of 0.05. The common genes are indicated by blue/white strips. The white area denotes similar genes in which three of four are the same. The unique genes are marked as color bands: 1) T3/HDF, orange; 2) T3/CMHDF, blue; 3) T5/MEF, red; 4) T3/CMMEF, green. B. The top 10 GeneGo canonical pathway maps.

The mRNAs expressed more than three folds of overall mean from T3/HDF and T3/CMHD, as well as T3/MEF and T3/CMMEF, cells were analyzed for GeneGo canonical pathway maps and GO process networks by using MetaCore Analytical Suite, and these four populations of hES cells abundantly expressed 560 common genes (Fig. 2A). T3/HDF and T3/CMHDF cells abundantly expressed 1,606 common genes, and 457 and 452 unique genes, respectively, whereas T3/MEF and T3/CMMEF cells abundantly expressed only 705 common genes, and 153 and 227 unique genes, respectively. It is of interest that the abundantly expressed genes (2063 & 2058) of T3/HDF and T3/CMHD cells are more than twice of those (858 & 932) of T3/MEF and T3/CMMEF cells (Fig. 2A).

The top 10 GeneGo canonical pathway maps of T3/HDF, T3/CMHDF, T3/MEF and T3/CMMEF cells are shown in Fig. 2B. The number 1 pathway of their 650 common genes is involved in development, that is, the role of Activin A in cell differentiation and proliferation, and another three of top 10 pathways are involved in cell adhesion (gap junctions, tight junctions and plasmin signaling). It may be further noted that the number 1 GO process network of their 650 common genes is also involved in cell adhesion (cell junctions), and four of top 10 GO process networks are involved in development (three neurogenesis) (Additional file 6: Fig. S3). The first two of the top 10 pathways of the 1256 similar genes among these four cell populations are cell adhesion (gap junctions and tight junctions) and the third pathway is regulation of metabolism (bile acids, glucose and lipid). The top three process networks of these 1256 similar genes are development (two neurogenesis and one hedgehog signaling). As to the differentially altered pathways of unique genes, the top three pathways in T3/HDF cells are cell adhesion (tight junction), immune response (neuroendocrine- macrophage connector) and signaling transduction (PKA signaling). The top three pathways in T3/CMHDF cells are development (role of activin A in cell differentiation and proliferation), cytoskeleton remodeling (neurofilament) and immune response (neuroendocrine-macropphage connector). The top three pathways in T3/MEF cells are cell adhesion (gap junctions), cytoskeleton remodeling (neurofilament) and regulation of metabolism (bile acids, glucose and lipid). The top two pathways in T3/CMMEF cells are cytoskeleton remodeling (neurofilament) and cell adhesion (tight junctions).

### Expression profiling of miRNAs

**Figure 3:**
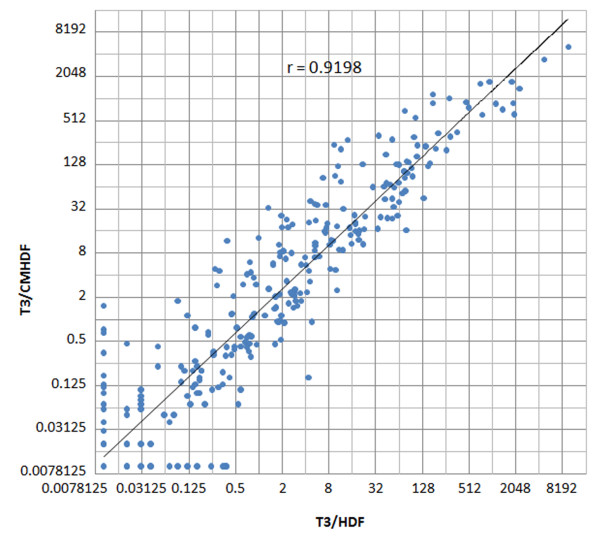
**Scatter plot and correlation analysis of miRNAs between T3/HDF and T3/CMHDF cells**. The scatter plot was graphed on log2 scale.

The expression profiles of 365 human miRNAs in T3/HDF and T3/CMHDF cells were quantitated using TaqMan miRNA Assays as described previously [5,6,9,10], and the expression level of each miRNA was indicated as folds over U6 snRNA. The average values of triplicate analyses and fold-changes for 365 miRNAs from these two different cell populations are given in Additional file 7: Table S3. The Pearson correlation coefficient of r = 0.9198 between T3/HDF and T3/CMHDF cells indicates their similar miRNA expression profiles (Fig. 3). The expression levels and fold-changes of 35 most abundantly expressed miRNAs of T3/HDF and T3/CMHDF, as well as those of 31 miRNAs of T3/MEF and T3/CMMEF, cells are summarized in Table 2. These results indicate that nine hES cell-specific miRNAs (miR-302a, 302b, 302c, 302 d, 367, 371, 372, 373 & 200c) were abundantly expressed in T3/HDF and T3/CMHDF cells, and that miR-367 and miR-373 had little more than 2-fold variations between these two cell populations. In addition, eleven other miRNAs (miR-26a, 31, 29a, 125b, 15b, 24, 125a, 21, 26b, 140 & 214) appeared to express more than 2-folds in T3/CMHDF compared with T3/HDF cells. It may also be noted that the miRNA data of T3/MEF and T3/CMMEF cells were previously determined using the set of 250 miRNAs in which miR-302a, 302b, 302c and 373 were not included, and that very similar expression profiles of miRNAs (r = 0.9624) between T3/MEF and T3/CMMEF cells were also found previously [6]. No miRNA with more than 2-fold variation was found between the 31 abundantly expressed miRNAs of T3/MEF and T3/CMMEF cells (Table 2).

**Table 2:** The expression levels and fold-changes of 35/31 most abundantly expressed miRNAs.

35 miRNAs	T3/HDF	T3/CMHDF	T3HDF/T3CMHDF	T3CMHDF/T3HDF	T3/MEF	T3/CMMEF	T3MEF/T3CMMEF	T3CMMEF/T3MEF	Specificity
hsa-miR-302b	9750.23	5140.91	1.90	0.53					hES
hsa-miR-302c	4723.51	3503.58	1.35	0.74					hES
hsa-miR-302a	2298.03	1399.09	1.64	0.61					hES
hsa-miR-367	1885.77	879.76	2.14	0.47	136.63	207.63	0.66	1.52	hES
hsa-miR-302d	1389.34	728.12	1.91	0.52	205.43	206.53	0.99	1.01	hES
hsa-miR-372	935.33	1743.19	0.54	1.86	27.97	15.65	1.79	0.56	hES
hsa-miR-200c	357.72	352.97	1.01	0.99	27.76	28.94	0.96	1.04	hES
hsa-miR-371	81.03	142.38	0.57	1.76	3.27	1.88	1.74	0.57	hES
has-miR-373	59.68	130.57	0.46	2.19					hES
hsa-miR-19b	1812.71	1723.10	1.05	0.95	366.63	319.07	1.15	0.87	
hsa-miR-20a	1128.35	876.96	1.29	0.78	353.83	327.13	1.08	0.92	
hsa-miR-222	753.37	617.30	1.22	0.82	287.47	195.53	1.47	0.68	
hsa-miR-26a	720.75	1617.54	0.45	2.24	199.23	133.67	1.49	0.67	
hsa-miR-16	502.41	774.74	0.65	1.54	339.10	360.13	0.94	1.06	
hsa-miR-92	472.46	913.07	0.52	1.93	203.40	200.47	1.01	0.99	
hsa-miR-93	292.52	309.16	0.95	1.06	153.10	144.93	1.06	0.95	
hsa-miR-31	282.45	1023.75	0.28	3.62	456.80	402.70	1.13	0.88	
hsa-miR-19a	260.70	201.61	1.29	0.77	43.74	63.94	0.68	1.46	
hsa-miR-130a	205.47	342.28	0.60	1.67	82.71	77.60	1.07	0.94	
hsa-miR-106b	188.85	213.30	0.89	1.13	31.72	29.19	1.09	0.92	
hsa-miR-29a	175.75	1171.80	0.15	6.67	143.60	124.77	1.15	0.87	
hsa-miR-125b	173.24	887.19	0.20	5.12	609.50	465.27	1.31	0.76	
hsa-miR-20b	160.19	133.91	1.20	0.84	45.07	32.02	1.41	0.71	
hsa-miR-30c	141.09	227.92	0.62	1.62	41.01	37.61	1.09	0.92	
hsa-miR-15b	110.35	235.37	0.47	2.13	19.82	21.89	0.91	1.10	
hsa-miR-30b	108.77	166.05	0.66	1.53	56.51	47.56	1.19	0.84	
hsa-miR-24	103.25	558.47	0.18	5.41	376.70	265.83	1.42	0.71	
hsa-miR-125a	99.95	308.23	0.32	3.08	267.10	168.33	1.59	0.63	
hsa-miR-186	95.26	89.09	1.07	0.94	26.08	20.02	1.30	0.77	
hsa-miR-221	84.51	137.99	0.61	1.63	254.57	154.90	1.64	0.61	
hsa-miR-21	75.40	688.55	0.11	9.13	80.24	64.43	1.25	0.80	
hsa-miR-191	73.55	105.10	0.70	1.43	30.65	24.64	1.24	0.80	
hsa-miR-26b	63.65	128.36	0.50	2.02	24.95	27.72	0.90	1.11	
hsa-miR-140	51.92	282.36	0.18	5.44	208.00	113.27	1.84	0.54	
hsa-miR-214	35.17	320.57	0.11	9.11	269.57	212.77	1.27	0.79	

### Protein patterns of 2D-gel analysis

**Figure 4:**
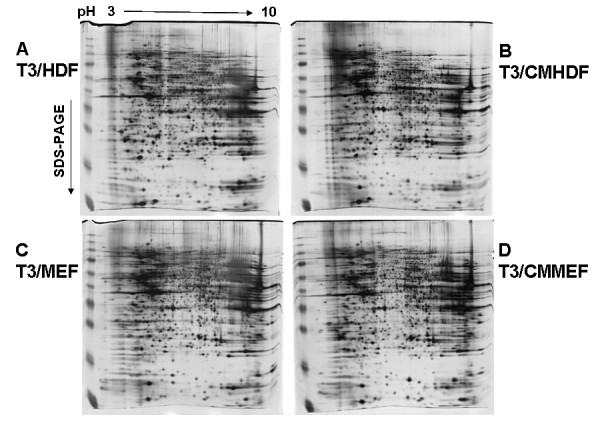
**Protein patterns on 2D-gel of T3/HDF, T3/CMHDF, T3/MEF and T3/CMMEF cells**.

The total soluble proteins extracted from T3/HDF and T3/CMHDF, as well as T3/MEF and T3/CMMEF, cells were separated on 2D-gels, and the silver staining patterns of protein spots from these four hES cell populations appeared to be very similar (Fig. 4). The similarities of protein spot patterns among these four 2D-gels were analyzed using ImageMaster (Additional files 8, 9,10 and 11: Fig. S4A, B, C, D), and their results are indicated in Table 3. A total of approximately 1627 spots (from 1566 to 1698) were separately detected, and approximately 1161 spots (71.42%) were matched among these four cell populations. It may be noted that the ranking orders of similarities among these four comparisons of protein spots were found to be the same to those of correlation coefficients of mRNAs (Fig. 1) and that the correlation coefficient (R) between % protein match spots and correlation coefficient (r) of mRNAs (Table 3) was found to be 0.8122 (Additional file 12: Fig. S5). In other words, the similarities of protein expression among these four cell populations were consistent with those of mRNA expression, although the extents of their protein similarities were smaller than those of mRNAs. The comparison of both protein spots and mRNA levels between T3/MEF and T3/CMMEF cells exhibited the most similarity, while that of T3/HDF and T3/MEF cells had lowest similarity.

**Table 3:** Similarities among expressions of protein spots and mRNAs of T3/HDF, T3/CMHDF, T3/MEF and T3/CMMEF cells.

Comparisons	Cells	No. of total spots	No. of match spots	% of match spots	Average of % match spots	* Corr. coeff. (r) of mRNAs
A	T3/HDF	1698	1176	69.26%	72.18%	0.9829
	T3/CMHDF	1566	1176	75.10%		
						
B	T3/MEF	1584	1192	75.25%	73.51%	0.9934
	T3/CMMEF	1661	1192	71.76%		
						
C	T3/HDF	1698	1114	65.61%	67.97%	0.9422
	T3/MEF	1584	1114	70.33%		
						
D	T3/CMHDF	1566	1161	74.14%	72.02%	0.9513
	T3/CMMEF	1661	1161	69.90%		

**Overall mean**		1627	1161		71.42%	0.9675

* Data are from Fig. 1.

## Discussion

The hES-T3 cell line with normal female karyotype, one of five hES cell lines derived in our laboratory [4], was used to differentiate into autogeneic fibroblast-like cells (T3HDF) as feeder to support the undifferentiated growth of hES-T3 cells (T3/HDF) for 14 passages [5] according to the previously published procedure [3]. Stojkovic et al. [3] reported that the hES cells cultured on autogeneic feeder and Matrigel in the presence of autogeneic conditioned medium for 44 and 14 passages, respectively, still maintained normal karyotype and expressed hES markers such as TRA-1-60, SSEA-4 and GTCM-2. This autogeneic feeder system was further shown to permit continuous growth of pluripotent hES cells as demonstrated by the formation of teratoma in SCID mice and in vitro differentiation. In this investigation, a feeder-free culture on Matrigel in medium conditioned by these autogeneic feeder cells (T3HDF) was established to maintain the undifferentiated growth of hES-T3 cells (T3/CMHDF) for 8 passages. The gene expression profiles of mRNAs, miRNAs and proteins among the undifferentiated T3/HDF, T3/CMHDF, T3/MEF and T3/CMMEF cells were shown to be very similar. In recent years, many improvements on standard MEF culture have been reported to develop xeno-free culture systems of hES cells for future clinical applications [11-17]. To our knowledge, this investigation is the first report that systematically compared and demonstrated the similar expression profiles of mRNAs, miRNAs and proteins among different feeders and conditioned media. However, many more passages (i.e. 20) of the undifferentiated growth of hES-T3 cells on autogeneic T3HDF feeder and feeder-free on Matrigel in the T3HDF conditioned medium should be carried out and their differentiation capacities (pluripotencies) should also be demonstrated using formation of embryoid bodies in vitro and/or teratoma in SCID mice in the future investigation.

The abundantly expressed genes of T3/HDF, T3/CMHD, T3/MEF and T3/CMMEF cells were found to play prominent roles in signaling pathways and GO process networks. Three of the top 10 GeneGo canonical pathway maps and four of the top 10 GO process networks of the common and/or similar genes among these four cell populations were involved in development. Their number 1 pathway was the role of Activin A in cell differentiation and proliferation, and the importance of Activin/Nodal/TGFβ family members in the maintenance of pluripotency of hES cells is widely established [18-20]. Among these common and/or similar genes, cell adhesion was also involved in three of the top 10 GeneGo canonical pathway maps and two of the top 10 GO process networks. However, the abundantly differentially expressed genes of T3/HDF and T3/MEF cells grown on feeder appeared to play important roles in cell adhesion (tight junctions and gap junctions), while those of T3/CMHDF and T3/CMMEF cells grown on feeder-free Matrigel in their conditioned medium had important effects on cytoskeleton remodeling. The fact that the many more genes were found to be expressed abundantly in T3/HDF and T3/CMHDF cells compared with T3/MEF and T3/CMMEF cells may indicate that autogeneic feeder cells and their conditioned medium were better suitable for the undifferentiated growth of hES cells than those of MEF. It is also of interest that galanin and galectin 1 were the most abundantly expressed genes in T3/HDF and T3/CMHDF cells, respectively. Galanin is a neuropeptide with important central nervous system actions (in particular its proposed role in Alzheimer's disease) [21]. The galectin-1 protein has been reported to have many diverse biological functions [22]. The specific roles of galanin and galectin-1 proteins in T3/HDF and T3/CMHDF cells remain to be investigated.

The miRNAs, a class of noncoding small RNAs that participate in the post-transcriptional regulation of gene expression, have been shown to play key roles in maintenance of the undifferentiated and pluripotent state as well as differentiation and lineage commitment of embryonic stem cells [23]. As demonstrated previously [24-27], the miR-302/367 cluster on chromosome 4 and miR-371/372/373 cluster on chromosome 19 were extremely abundantly expressed in undifferentiated hES-T3 cells grown on T3HDF feeder (T3/HDF) and feeder-free Matrigel in T3HDF-conditioned medium (T3/CMHDF), as well as MEF feeder (T3/MEF) and feeder-free Matrigel in MEF-conditioned medium (T3/CMMEF). The members of these two clusters share a consensus seed sequence and their targeted genes have overlapping functions [5,26,27]. The extremely abundant expression of hES cell-specific miR-302/367 and miR-371/372/373 clusters also indicated the very high proportion of undifferentiated hES cells present in these four cell populations. Recently, we reported that the expression of hES cell-specific miRNAs miR-302 d, miR-372 and miR-367 and miR-200c, as well as miR-199a, were strongly up-regulated by activin A [6]. It should also be noted that the large variations between the miRNA expression levels of T3/HDF and T3/CMHDF cells and those of T3/MEF and T3/CMMEF cells were most likely due to the different platforms (containing 365 and 250 miRNAs, respectively) used.

The soluble proteins of T3/HDF, T3/CMHDF, T3/MEF and T3/CMMEF cells were separated on 2D-gels, and their patterns of protein spots appeared to be very similar. The extents of protein similarities among these four cell populations appeared to be smaller than those of mRNAs, and these results may be due to the more variations of proteins because of post-translational modifications and/or technical variations among different 2D-gels. In the future studies, the proteins, which will be extracted using the classic RIPA buffer (Pierce) to obtain more proteins from the cells, from two different cell populations will be first labeled separately with Cy3 and Cy5 dyes, and then pooled together for comparison on a single 2D-gel in order to detect more accurately their similarities and differences. The different protein spots among these four cell populations will be identified using tandem mass spectrometry. It will be of interest if these different proteins were found to be in common with their unique genes detected in mRNA profiling. Although the proteomes of hES cells have previously been reported [28,29], the quantitative comparison between proteomes of hES-T3 cells and their differentiated fibroblasts (T3HDF) is being investigated in our laboratory. Our preliminary results (S.S.-L.Li, et al. unpublished) indicate that many of abundantly differentially expressed proteins are found to be heterogeneous nuclear ribonucleoproteins (hnRNPs). This finding of abundant hnRNP proteins is consistent with the facts that hES cells exhibit high ratio of nucleus to cytoplasm [1] and the hnRNP proteins are among the most abundant proteins in nucleus [30]. As to the proteome of T3DF cells, the abundantly differentially expressed proteins include several glycolytic enzymes such as L-lactate dehydrogenase A(M), and this observation is also consistent with the more anaerobic metabolism of fibroblasts.

## Conclusion

The hES-T3 cell line was previously used to differentiate into autogeneic fibroblast-like cells (T3HDF) as feeder to support the undifferentiated growth of hES-T3 cells (T3/HDF). In this investigation, a feeder-free culture on Matrigel in hES medium conditioned by these autogeneic feeder cells (T3HDF) was established to maintain the undifferentiated growth of hES-T3 cells (T3/CMHDF). The gene expression profiles of mRNAs, microRNAs and proteins between the undifferentiated T3/HDF and T3/CMHDF cells were shown to be very similar, and their expression profiles were also found to be similar to those of T3/MEF and T3/CMMEF cells grown on MEF feeder and feeder-free Matrigel in MEF-conditioned medium, respectively. The undifferentiated state of T3/HDF and T3/CMHDF, as well as T3/MEF and T3/CMMEF, cells was evidenced by the very high expression levels of "stemness" genes, as well as hES cell-specific miR-302/367 and miR-371/372/373 clusters, and low expression levels of differentiation markers of ectoderm, mesoderm and endoderm in addition to the strong staining of OCT4 and NANOG. Thus, the T3HDF feeder and T3HDF-conditioned medium were able to support the undifferentiated growth of hES cells, and they would be useful for drug development and toxicity testing in addition to the reduced risks of xenogeneic pathogens when used for medical applications such as cell therapies.

## Database and accession number

The original data obtained from Affymetrix human genome U133 plus 2.0 GeneChip have been deposited to NCBI database, and the GEO series number is GSE19902.

## Abbreviations

hES: human embryonic stem cells; miRNAs: microRNAs; MEF: mouse embryonic fibroblasts; T3/MEF: hES-T3 cells grown on MEF feeder; T3/CMMEF: hES-T3 cells grown on feeder-free Matrigel in MEF-conditioned medium; T3HDF: hES-T3 differentiated fibroblast-like cells; T3/HDF: hES-T3 cells grown on T3HDF feeder; T3/CMHDF: hES-T3 cells grown on feeder-free Matrigel in T3HDF-conditioned medium.

## Competing interests

The authors declare that they have no competing interests.

## Authors' contributions

ZYT established T3HDF cells and cultured T3/HDF, T3/CMHDF, T3/MEF and T3/CMMEF cells. SS did bioinformatic analyses. S-LY supervised the determination of both miRNAs and mRNAs. CHC did protein 2D-gel analysis, SS-LL designed the experiments, analyzed results and wrote the manuscript. All authors read and approved the final manuscript.

## Authors' information

ZYT, MS, Research Assistant; SS, Ph.D., Assistant Professor; S-LY, Ph.D., Associate Professor; CHC, MS, Research Assistant; SS-LL, Ph.D., Chair Professor.

## Supplementary Material

Additional file 1 **Fig. S1**. The OCT4 and NANOG staining of T3/HDF and T3/CMHDF, as well as T3/MEF and T3/CMMEF, cells. T3/HDF and T3/CMHDF cells were grown on T3HDF feeder and feeder-free Matrigel in T3HDF-conditioned medium for 14 and 8 passages, respectively. The T3/MEF and T3/CMMEF cells were grown on MEF feeder and feeder-free on Martigel in MEF-conditioned medium for 14 and 12 passages, respectively. Click here for file

Additional file 2 **Table S1A**. 102 genes abundantly differentially expressed in T3/HDF cells. Click here for file

Additional file 3 **Table S1B**. 84 genes abundantly differentially expressed in T3/CMHDF cells. Click here for file

Additional file 4 **Fig. S2**. Hierachical clustering (left) and principle component analysis (right) of all microarray data. Click here for file

Additional file 5 **Table S2**. The expression levels and fold-changes of 21 "stemness" genes and 9 differentiation markers in T3/HDF, T3/CMHDF, T3/MEF and T3/CMMEF cells. Click here for file

Additional file 6 **Fig. S3**. The top 10 GO process networks of the abundantly expressed genes among T3/HDF, T3/CMHDF, T3/MEF and T3/CMMEF cells. The common genes are indicated by blue/white strips. The white area denotes similar genes in which three of four are the same. The unique genes are marked as color bands: T3/HDF, orange; T3/CMHDF, blue; T5/MEF, red; T3/CMMEF, green. Click here for file

Additional file 7 **Table S3**. The expression levels and fold-changes of 365 miRNAs in T3/HDF and T3/CMHDF cells. Click here for file

Additional file 8 **Fig. S4A**. Comparison of protein spots on 2D-gels between T3/HDF and T3/CMHDF cells. Green, match spots; red, unmatch spots. Click here for file

Additional file 9 **Fig. S4B**. Comparison of protein spots on 2D-gels between T3/MEF and T3/CMMEF cells. Green, match spots; red, unmatch spots. Click here for file

Additional file 10 **Fig. S4C**. Comparison of protein spots on 2D-gels between T3/HDF and T3/MEF cells. Green, match spots; red, unmatch spots. Click here for file

Additional file 11 **Fig. S4D**. Comparison of protein spots on 2D-gels between T3/CMHDF and T3/CMMEF cells. Green, match spots; red, unmatch spots. Click here for file

Additional file 12 **Fig. S5**. The relationship between the similarities (%) of protein match spots and correlation coefficients (r) of mRNAs. Click here for file
