# Supplementary figures and images for: A feeder-free culture using autogeneic conditioned medium for undifferentiated growth of human embryonic stem cells: Comparative expression profiles of mRNAs, microRNAs and proteins among different feeders and conditioned media
Source: BMC Cell Biol. 2010 Oct 12;11:76. doi: 10.1186/1471-2121-11-76 (PMC2958897; doi:10.1186/1471-2121-11-76)

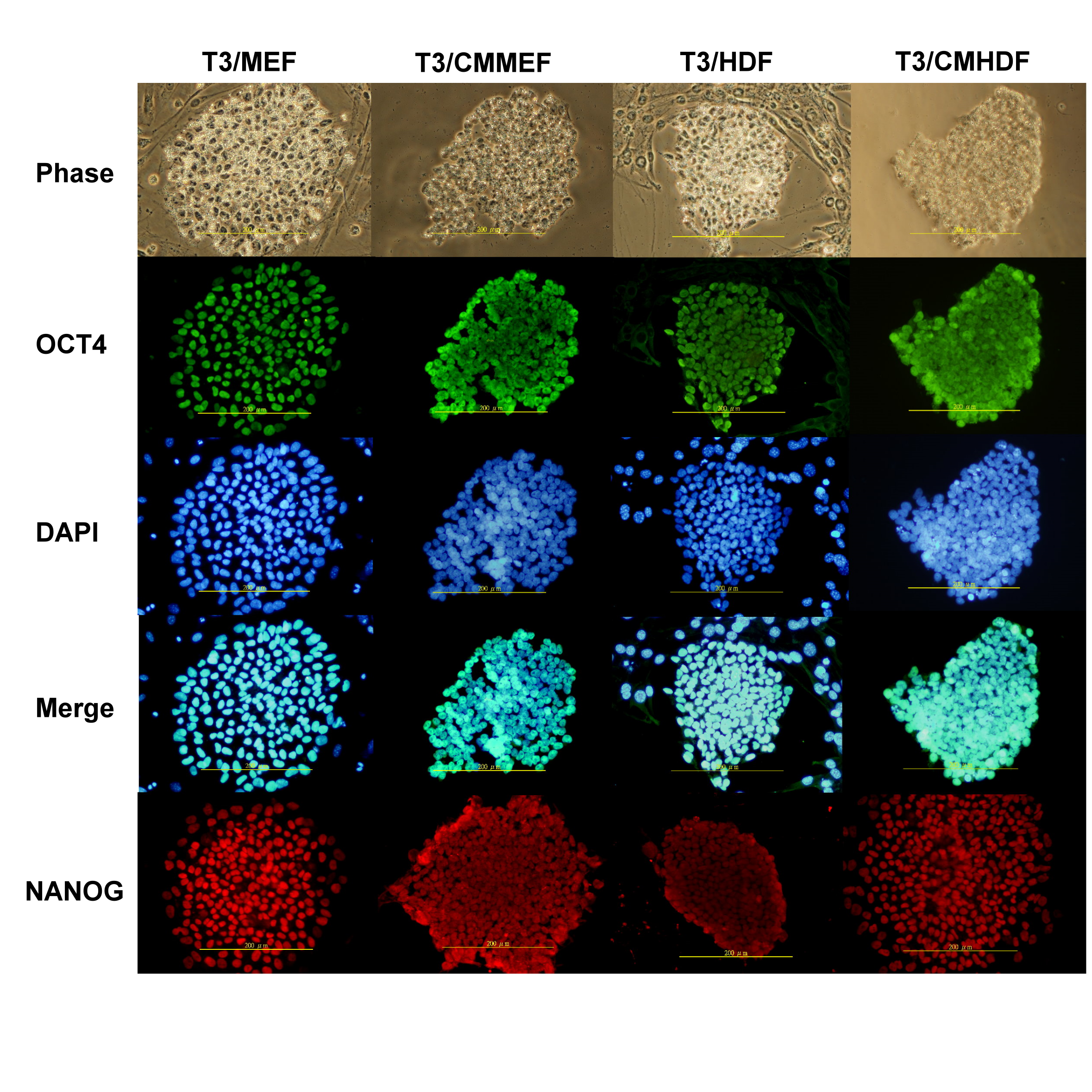

Supplement: Additional file 1 — Fig. S1. The OCT4 and NANOG staining of T3/HDF and T3/CMHDF, as well as T3/MEF and T3/CMMEF, cells. T3/HDF and T3/CMHDF cells were grown on T3HDF feeder and feeder-free Matrigel in T3HDF-conditioned medium for 14 and 8 passages, respectively. The T3/MEF and T3/CMMEF cells were grown on MEF feeder and feeder-free on Martigel in MEF-conditioned medium for 14 and 12 passages, respectively. [file 1471-2121-11-76-S1.TIFF]

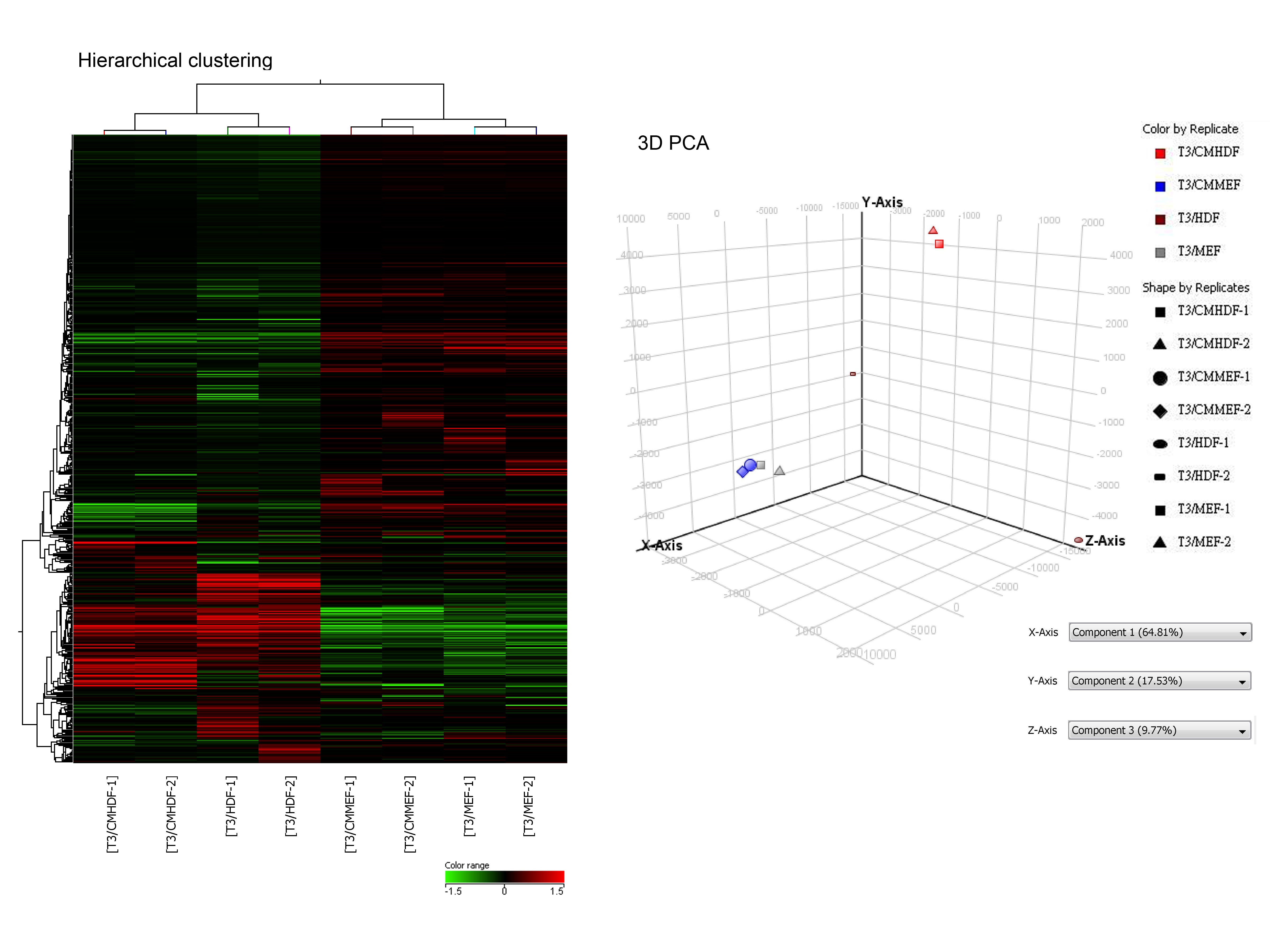

Supplement: Additional file 4 — Fig. S2. Hierachical clustering (left) and principle component analysis (right) of all microarray data. [file 1471-2121-11-76-S4.JPEG]

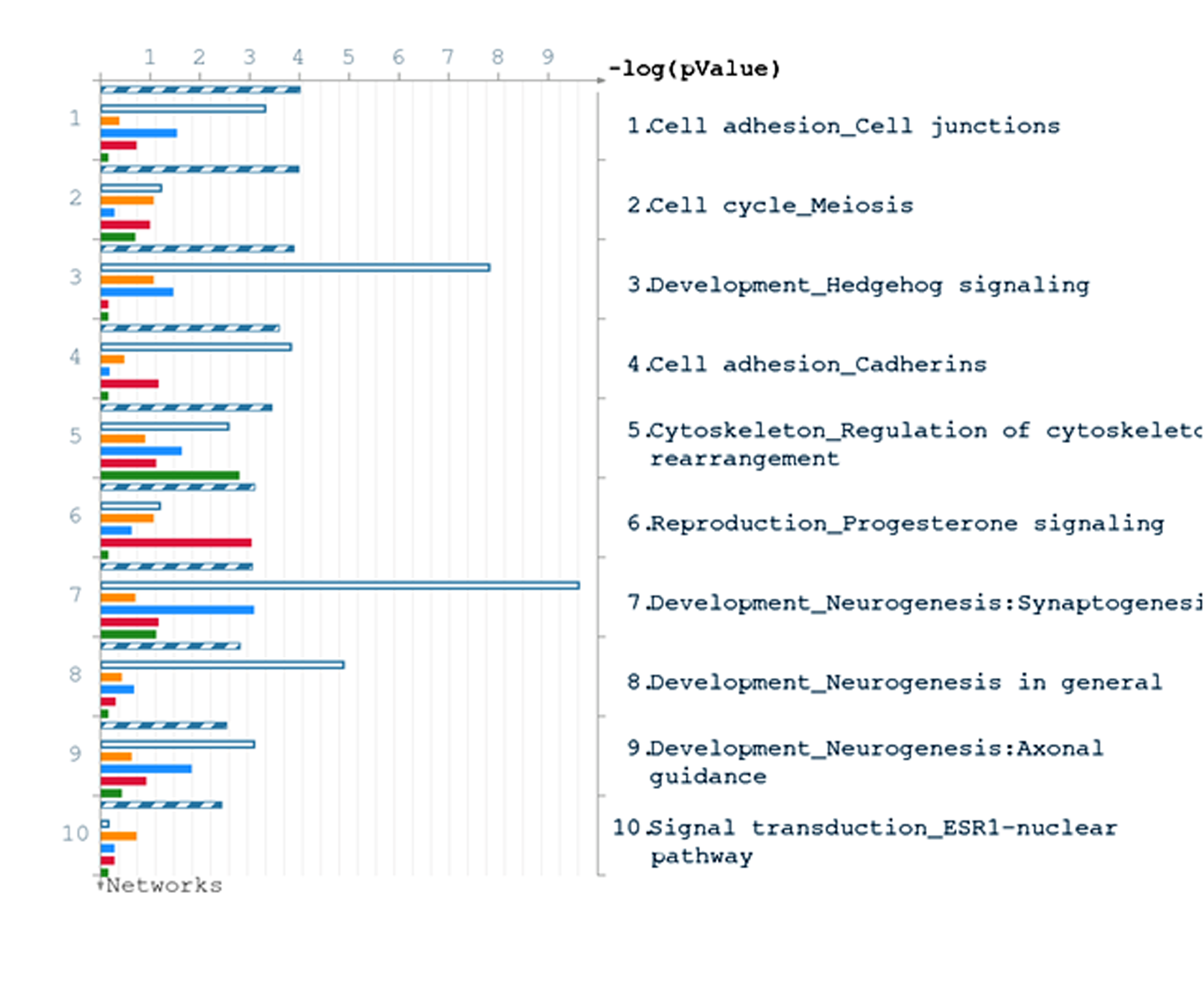

Supplement: Additional file 6 — Fig. S3. The top 10 GO process networks of the abundantly expressed genes among T3/HDF, T3/CMHDF, T3/MEF and T3/CMMEF cells. The common genes are indicated by blue/white strips. The white area denotes similar genes in which three of four are the same. The unique genes are marked as color bands: T3/HDF, orange; T3/CMHDF, blue; T5/MEF, red; T3/CMMEF, green. [file 1471-2121-11-76-S6.TIFF]

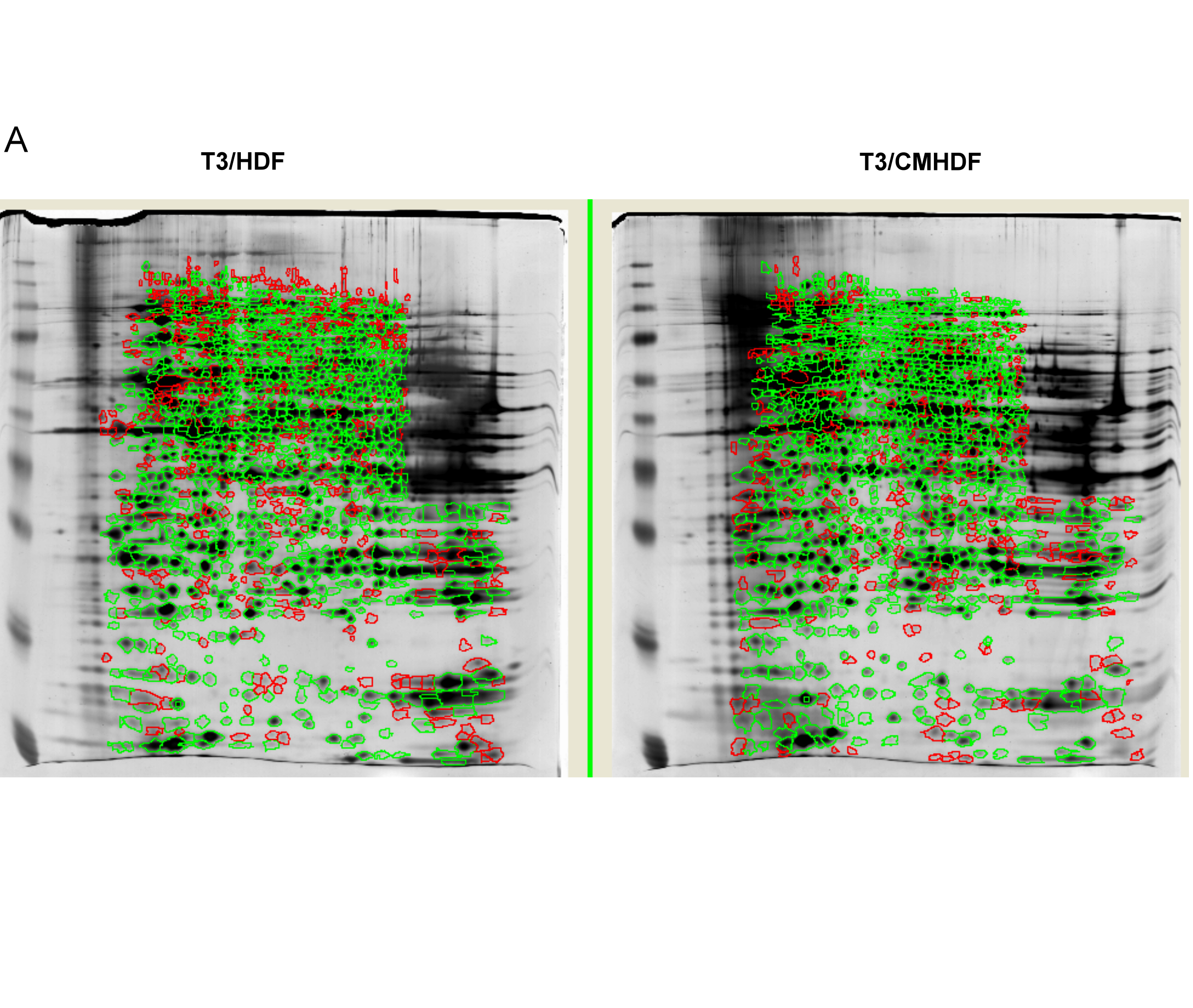

Supplement: Additional file 8 — Fig. S4A. Comparison of protein spots on 2D-gels between T3/HDF and T3/CMHDF cells. Green, match spots; red, unmatch spots. [file 1471-2121-11-76-S8.TIFF]

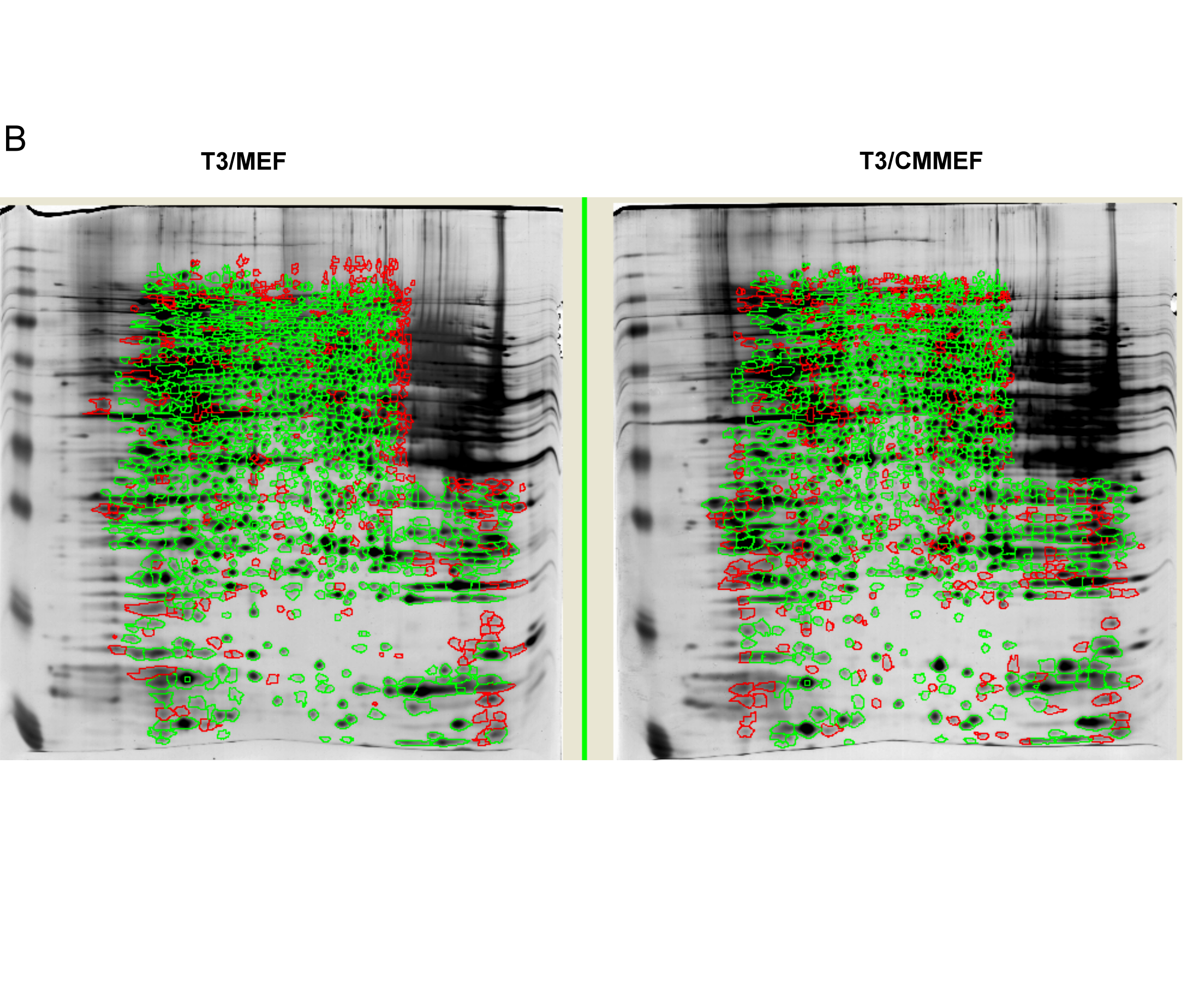

Supplement: Additional file 9 — Fig. S4B. Comparison of protein spots on 2D-gels between T3/MEF and T3/CMMEF cells. Green, match spots; red, unmatch spots. [file 1471-2121-11-76-S9.TIFF]

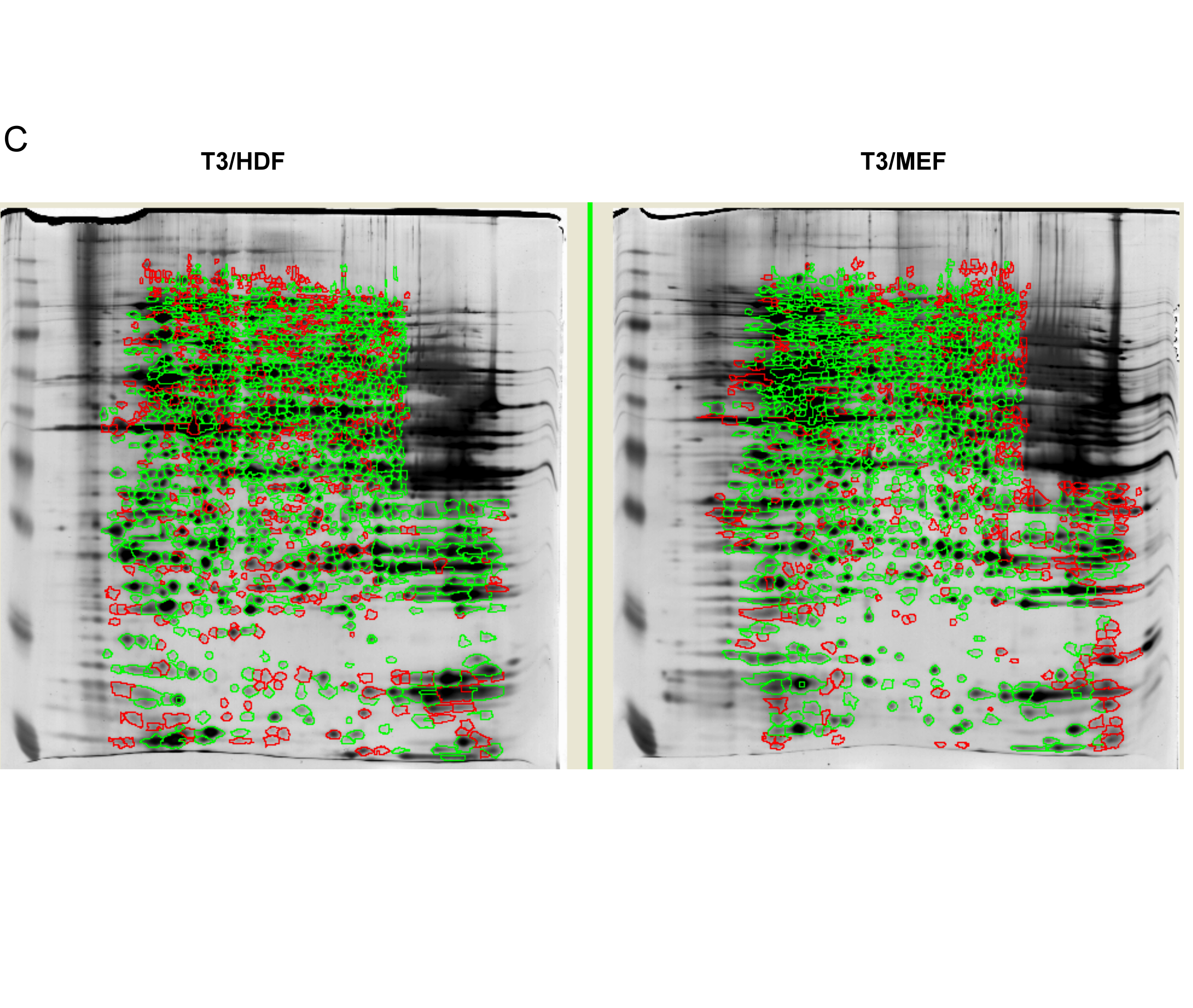

Supplement: Additional file 10 — Fig. S4C. Comparison of protein spots on 2D-gels between T3/HDF and T3/MEF cells. Green, match spots; red, unmatch spots. [file 1471-2121-11-76-S10.TIFF]

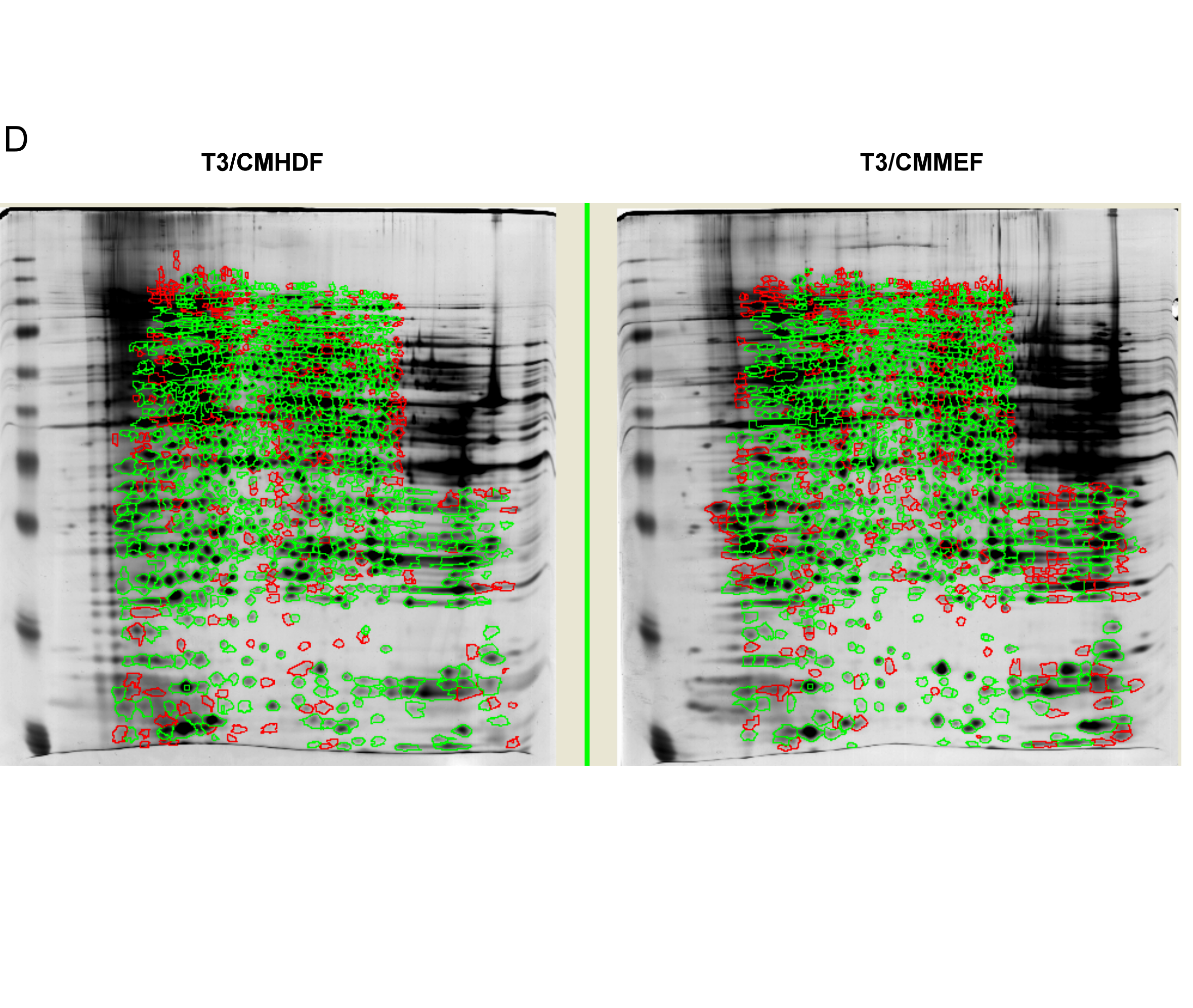

Supplement: Additional file 11 — Fig. S4D. Comparison of protein spots on 2D-gels between T3/CMHDF and T3/CMMEF cells. Green, match spots; red, unmatch spots. [file 1471-2121-11-76-S11.TIFF]

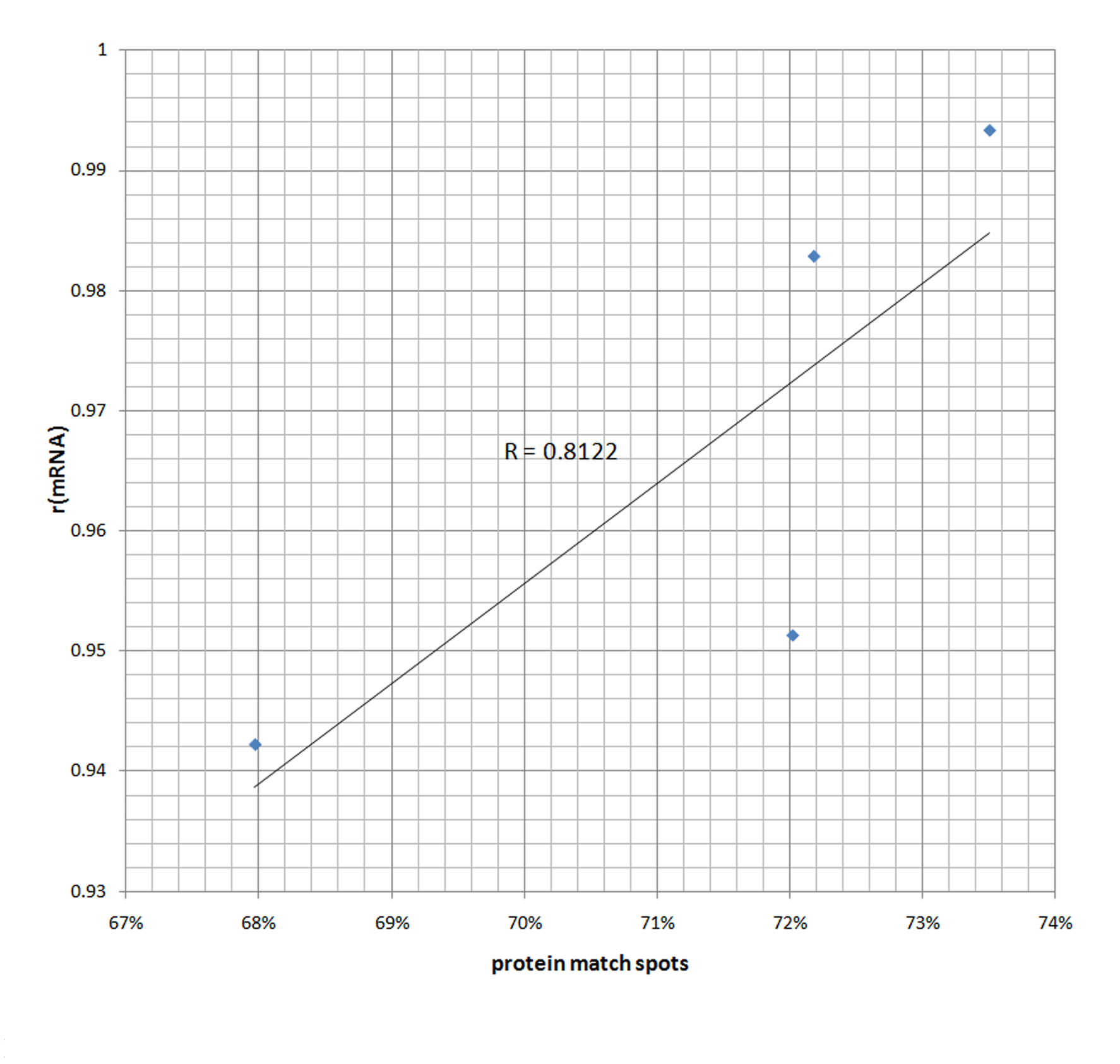

Supplement: Additional file 12 — Fig. S5. The relationship between the similarities (%) of protein match spots and correlation coefficients (r) of mRNAs. [file 1471-2121-11-76-S12.TIFF]
